# Bibliography

**Published:** 1904-12

**Authors:** 


### Bibliography.

Essig and Koenig's Dental Metallurgy. A Manual for the Use of Dental Students and Practitioners. By Charles J. Essig, M.D., D.D.S., formerly Professor of Mechanical Dentistry and Metallurgy in the Dental Department of the University of Pennsylvania, and Augustus Koenig, B.S., M.D., Demonstrator of Metallurgy in the Dental Department of the University of Pennsylvania, etc. New (fifth) edition, revised and enlarged. In one 12mo volume of three hundred and eighteen pages, with seventy-six engravings. Lea Brothers & Co., Philadelphia and New York, 1904.

This publication, originally prepared by Dr. Charles J. Essig, and now, in its fifth edition, revised by Augustus Koenig, B.S., M.D., still remains as the most satisfactory text-book on metallurgy.

When Professor Essig originally issued this book it was regarded as of more value to practitioners than to students, for the subject was indifferently taught in dental colleges; in fact, it was not thought of sufficient importance to have it introduced as a separate study. This book changed all this, for through its quiet influence courses in metallurgy have been established universally.

It requires no argument here to demonstrate the importance of this study to the dentist,—in fact, a knowledge of this to some extent, at least, is demanded of every practitioner, and he who failed to secure it at college will find it to his advantage to follow Essig and Koenig through this book.

Thirty-two new cuts have been added to this edition, which with those previously in place increase the illustrations to seventy-six.

The fact that this has been translated and used as a text-book in foreign colleges is a sufficient recommendation, and an assurance that under its present able editor it will remain as the standard publication on the subject.

Normal Histology. By Edward K. Dunham, Ph.B., M.D., Professor of General Pathology, Bacteriology, and Hygiene in the University and Bellevue Hospital Medical College, New York. Third edition, revised and enlarged. Illustrated with two hundred and sixty engravings. Lea Brothers & Co., New York and Philadelphia, 1904.

The fact that a work on any subject has reached a third edition is sufficient evidence that it has met a demand. This book, however, carries its own credentials with it and must necessarily command attention, for it has been prepared by an experienced teacher in this direction.

The author begins the study of histology by " First, the conception of the cell as the active constituent of tissues; second, the general rule that the elementary tissues are composed of cells and intercellular substances, and that their differences depend upon the proportions or characters of those constituents; third, that the structural details of the different tissues are intimately correlated to their usefulness—*i.e.*, that function and structure are mutually dependent upon each other, being but two aspects of a single device or arrangement; and fourth, that the ability of tissues to perform active functions is, in the main, roughly proportional to the number or size of the cells entering into their structure."

With this as a basis of work the author has prepared an exceedingly valuable text-book on " Normal Histology" that cannot fail to have a distinct value to students of medicine and also to students of dentistry in their general histological work.

The author fails, however, as all medical authors do fail, to meet the needs of the dental student in his special work. It is strange that medical teachers when called to explain anything dental immediately make it a fit subject for indifferent treatment. The author devotes just one page and a quarter to " The Teeth," with three illustrations, two showing development and one the tissues diagrammatically. The author says, " Only a brief description of the structures entering into the formation of the fully developed tooth can be given here." It seems to the writer that the development of teeth from the histologist's stand-point is as important and interesting as any portion of the organism, and the various tissues composing the teeth are of quite as much importance in the human economy as bone, to which the author devotes six pages.

Dental students can secure this knowledge elsewhere, but in other respects the book can be cordially recommended for their study in connection with their practical work in the laboratory.

THE HISTORY OF A HISTORY. Souvenir. Fourth International Dental Congress, St. Louis, Mo., August 29 to September 3, 1904.

This is a very interesting little volume giving a brief history of the foundation of the present *Dental Cosmos* and that of the *Dental News Letter* of 1847, and also the history of the house of S. S. White Dental Manufacturing Company since it was founded by Dr. Samuel S. White in 1844, at the age of twenty-two.

To one familiar, as the writer is, with every stage of progress made by this great house, from the time when Dr. White opened his first place of business on Seventh and Race Streets, Philadelphia, to the present time, when it has branches all over the world, this souvenir claims special interest. To the pleasure of recalling facts is added the feeling of mournful regret as we look into the face of the founder and that of his brother James to feel that they are no longer part of the active life of this world. It is something of a compensation to know, however, that the house and our able contemporary, the *Dental Cosmos,* have gone steadily forward, each step taken indicating, indirectly it may be, but still indicating the growth of the dental profession, with which during all these years they have been so intimately connected.

The temptation is, when examining such a souvenir, to allow the mind to travel back to the past and to the men connected with this house and with dentistry, but space does not permit the reminiscent mood.

Excellent pictures are given of all connected with the house at the present, as well as those of the past. These add much to the interest. From President Lewes, General Manager Gilbert, down to the branch managers, each and all tell a story of progress. The congratulations of the INTERNATIONAL DENTAL JOURNAL are extended.

General Catalogue of Medical Books. P. Blakiston's Son & Co., Philadelphia.

This is a very convenient pocket catalogue, giving title and price of the leading medical and dental books published. In this respect it seems to have been very carefully compiled, and with its interleaves will enable memoranda to be made for future reference. The question of price is one constantly made and not always readily answered. The firm of Blakiston have, therefore, given a really valuable compilation in small compass to the profession.

